# Case Report: Hypercalcemia as a manifestation of acute adrenal crisis precipitated by fluconazole use, and a review of the literature

**DOI:** 10.3389/fendo.2023.1168797

**Published:** 2023-05-18

**Authors:** Kuan Swen Choo, Jielin Yew, Eberta Jun Hui Tan, Troy Hai Kiat Puar

**Affiliations:** ^1^ Department of Endocrinology, Changi General Hospital, Singapore, Singapore; ^2^ Raffles Diabetes and Endocrine Centre, Raffles Medical Group, Singapore, Singapore

**Keywords:** anti-fungal, azole, adrenal insufficiency, iatrogenic Cushing’s, hemodialysis, hypothalamic-pituitary-adrenal axis, hypercalcemia

## Abstract

Acute adrenal crisis classically presents with vomiting, altered sensorium, and hypotension. We describe a unique case manifesting with severe hypercalcemia. Addisonian crisis was unusually precipitated by fluconazole use. We reviewed other reported cases and discuss the possible mechanisms of hypercalcemia in adrenal insufficiency. This 67-year-old man presented with fever, cough, and vomiting for 1 week and with anorexia and confusion for 3 weeks. He was hypotensive and clinically dehydrated. Investigations revealed left-sided lung consolidation, acute renal failure, and severe non–parathyroid hormone (PTH)–mediated hypercalcemia (calcium, 3.55mol/L; PTH, 0.81pmol/L). Initial impression was pneumonia complicated by septic shock and hypercalcemia secondary to possible malignancy. He received mechanical ventilation; treatment with intravenous fluids, inotropes, and hydrocortisone for septic shock; and continuous renal replacement therapy with low-calcium dialysate. Although hypercalcemia resolved and he was weaned off inotropes, dialysis, and hydrocortisone, his confusion persisted. When hypercalcemia recurred on day 19 of admission, early morning cortisol was <8 nmol/L, with low ACTH level (3.2 ng/L). Other pituitary hormones were normal. Hypercalcemia resolved 3 days after reinstating stress doses of hydrocortisone, and his mentation normalized. On further questioning, he recently received fluconazole for a forearm abscess. He previously consumed traditional medications but stopped several years ago, which may have contained glucocorticoids. He was discharged on oral hydrocortisone. Cortisol levels improved gradually, and glucocorticoid replacement was ceased after 8 years, without any recurrence of hypercalcemia or Addisonian crisis. Both hypercalcemia and adrenal insufficiency may present with similar non-specific symptoms. It is important to consider adrenal insufficiency in hypercalcemia of unclear etiology.

## 1 Introduction

Acute adrenal insufficiency, or Addisonian crisis, classically presents with nausea, vomiting, altered mental status, and hypotension. Severe hypercalcemia is a rare manifestation. Precipitants of Addisonian crises include concurrent infection, particularly gastrointestinal infections and physical or mental stress. We report a rare case of severe hypercalcemia presenting with acute confusion and shock, precipitated by iatrogenic adrenal insufficiency. The use of fluconazole inhibits adrenal steroidogenesis and aggravated pre-existing adrenal insufficiency, leading to acute adrenal crisis. We review previous reports of fluconazole-associated adrenal insufficiency and the pathophysiology of severe hypercalcemia with adrenal insufficiency.

## 2 Case description

In September 2010, this 67-year-old man presented with fever, productive cough, and vomiting for 1 week. This was associated with progressive weight loss, poor appetite, lethargy, and increasing confusion over the past 3 weeks. He did not have abdominal pain, chronic cough, night sweats, or altered bowel habit. There was no headache, palpitations, or diaphoresis.

His past medical history included hypertension, hyperlipidemia, and right inguinal hernia, which was operated on 10 years ago. Chronic medications included amlodipine, lisinopril, and simvastatin. He was not on corticosteroids, diuretics, lithium, antacids, calcium, or vitamin D supplementation. Four weeks prior, he underwent saucerization of a right forearm abscess and received a course of antimicrobials. He did not consume alcohol or recreational drug.

On examination, he was confused with a Glasgow Coma scale (GCS) of 14. He was febrile and hemodynamically unstable with blood pressure of 80/50 mmHg, heart rate of 80 beats per minute, and oxygen saturation of 99% on room air. He was clinically dehydrated. Cardiovascular, respiratory, and abdominal examination was unremarkable, with no focal neurological deficit or neck rigidity. He did not have any features of Cushing’s syndrome, and there were no hyperpigmented skin creases or buccal mucosa.

Initial investigations showed left upper lobe consolidation on chest radiograph and raised inflammatory markers. Computed tomography (CT) scan of the brain was unremarkable. Biochemical investigation revealed acute renal failure (creatinine at 407 µmol/L, from 84 µmol/L 3 weeks prior) and severe hypercalcemia of 3.55 mmol/L, which was deemed the likely cause of his altered sensorium. Intact parathyroid hormone (iPTH) was low at 0.81 pmol/L (reference: 1.3–7.6 pmol/L). Electrocardiogram revealed normal sinus rhythm with no shortened QTc interval. There was mild hyponatremia (serum sodium of 132 mmol/L) with normokalemia.

The initial impression was pneumonia complicated by septic shock and acute renal failure and hypercalcemia secondary to underlying malignancy in view of his loss of weight. He was immediately initiated on intravenous (IV) 0.9% sodium chloride drip and IV antibiotics (piperacillin/tazobactam and azithromycin) for healthcare-associated pneumonia. Despite 4.5 L of IV fluids, he had refractory hypotension and was put on ionotropic support, followed by IV hydrocortisone of 100 mg every 8 h as per conventional treatment of septic shock (1). He was intubated for airway protection in view of dropping GCS. Subsequently, he underwent continuous renal replacement therapy (CRRT) with low-calcium dialysate for acute renal failure with pulmonary congestion and persistent hypercalcemia.

After 5 days, his hemodynamics and renal function improved, and he was weaned off ionotropic support, hydrocortisone, and CRRT. Despite resolution of pneumonia and normalization of serum calcium, he remained persistently drowsy and febrile. Antimicrobial therapy was escalated to cover for presumptive meningoencephalitis with IV meropenem, vancomycin, and acyclovir. Magnetic resonance imaging (MRI) of his brain was unremarkable, and cerebrospinal fluid (CSF) study was negative for acid fast bacilli, fungal, bacteria, and neurotropic viruses, although electroencephalogram showed severe diffuse encephalopathy with no epileptiform activity. Although his fever resolved and overall condition improved, he remained confused when he was transferred out of the intensive care unit (ICU) on day 17 of admission.

### 2.1 Work-up for hypercalcemia

**Figure 1:**
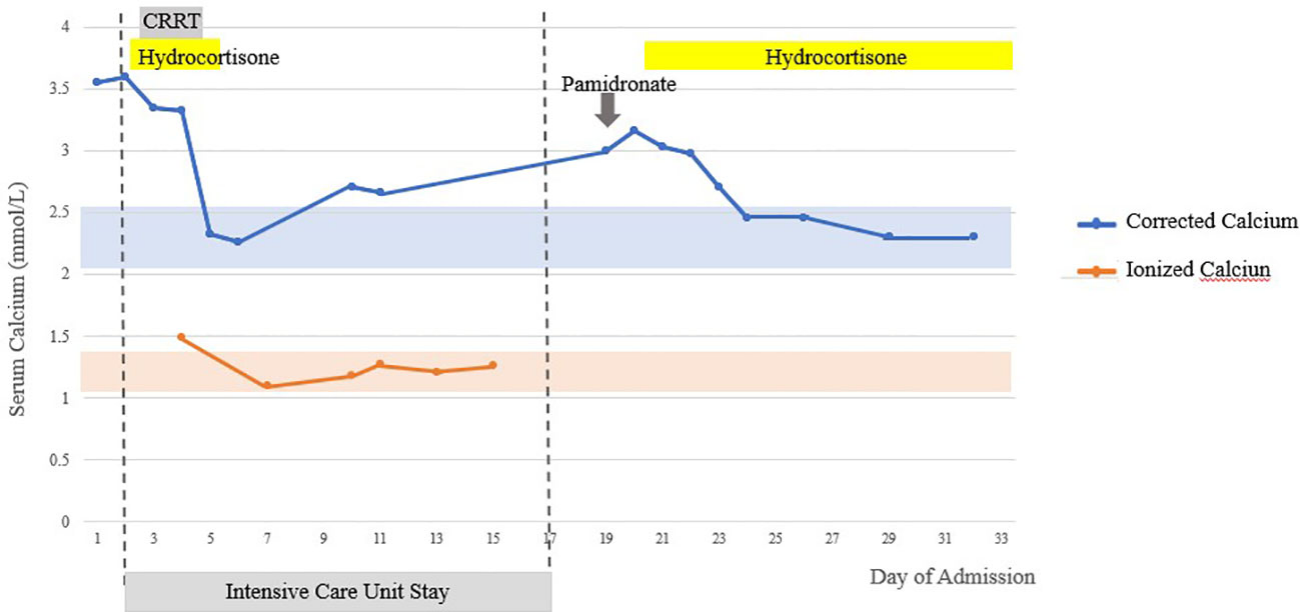
Serum-corrected calcium and ionized calcium trend inpatient. Shaded areas represent normal laboratory range.

His calcium levels (corrected for albumin and ionized calcium) returned to normal after IV fluids, hydrocortisone, and CRRT. Ionized calcium was monitored in ICU during CRRT that can alter total serum calcium, as well as hypoalbuminuria and acid base disturbances. Ionized calcium level remained normal for 12 days after cessation of CRRT and hydrocortisone. However, after transfer out of ICU, hypercalcemia recurred with peak corrected calcium of 3.16 mmol/L on day 19 of admission (**Figure 1**). He received IV NS hydration and IV pamidronate of 90 mg slow infusion on recurrence of hypercalcemia with little effect.

Initial serum phosphate was high normal at 1.62 mmol/L (reference: 0.65–1.65 mmol/L), and he was vitamin D–insufficient (25-hydroxyvitamin D at 22.6 µg/L). Severe PTH-independent hypercalcemia is most commonly secondary to hypercalcemia of malignancy, and this was consistent with his history of weight loss. However, there was no evidence of malignancy on CT thorax, abdomen, pelvis, and MRI brain. His alkaline phosphatase (ALP) was normal at 58 U/L (reference: 32–103 U/L), which suggested the absence of bone lesions, and myeloma panel was negative. There was no mediastinal enlargement, lymphadenopathy, or hepatosplenomegaly suggesting sarcoidosis or lymphoma on imaging. The endotracheal aspirate and CSF studies were negative for tuberculosis and fungi. Immobilization can cause hypercalcemia in the presence of high bone turnover conditions, but there was no prolonged immobilization prior to admission nor elevated ALP suggesting Paget’s disease of the bone or hyperthyroidism. Vitamin A level was sent and returned several weeks later, as low, at 0.2 mg/L (reference: 0.3–0.8 mg/L).

Although his hypotension and hyponatremia had resolved, a short Synacthen test was performed the following morning, with a blunted response: baseline undetecable, <8 nmol/L; and peak serum cortisol, 12 nmol/L. Serum cortisol had not been assessed prior to hydrocortisone initiation in the ICU. Immediately, IV hydrocortisone of 50 mg every 8 h was reinitiated. Adrenocorticotropic hormone (ACTH) level was low at 3.2 ng/L (reference: 10–60 ng/L), consistent with secondary adrenal insufficiency. His other pituitary hormones were normal: free T4, 13.7 pmol/L; thyroid-stimulating hormone, 2.84 mIU/L; Insulin-like Growth Factor-1 (IGF-1), 62.6 ng/ml; prolactin, 141.8 mIU/L; and total testosterone, 15.4 nmol/L. His calcium level improved markedly after 3 days of IV hydrocortisone, and it was converted to oral hydrocortisone of 20 mg every 8 h and subsequently tailed down to 20 mg daily in divided doses. His altered mentation resolved more gradually, and, by 7 days, he made a complete recovery.

On further questioning, he revealed that he previously consumed “Jamu” (traditional Malay medication) to improve vitality but had stopped 5 years prior to admission. “Jamu” are often made up from blends of herbal ingredients. As they are unregulated, they may contain unusually high amount of corticosteroids (2). There was no other exogenous steroid consumption or application. However, he was prescribed oral fluconazole of 200 mg twice daily for 4 weeks for his forearm abscess, as cultures showed “unspecified mould”. He had been consuming this up till this admission.

There was no history of previous head trauma, surgery, or radiotherapy. MRI pituitary gland was normal with no adenoma, stalk thickening, or loss of T2 bright spot suggesting lymphocytic hypophysitis. One year later, although all the other anterior pituitary hormones remained normal, serum cortisol remained low, at 15 nmol/L, whereas ACTH increased to 78.2 ng/L, suggesting recovery process of the hypothalamic-pituitary-adrenal (HPA) axis from chronic suppression with exogenous steroid. Serum aldosterone was normal at 17 ng/dl, with plasma renin activity at 4.5 ng/ml/h. Furthermore, previous CT imaging of his abdomen did not show any adrenal masses, hemorrhage, or atrophy.

### 2.2 Follow-up and outcomes

We established a final diagnosis of adrenal insufficiency secondary to HPA axis suppression from chronic traditional medication use, with Addisonian crisis and hypercalcemia precipitated by fluconazole use. Whereas his ACTH increased after the first year, his cortisol levels rose more gradually. After 8 years, glucocorticoid replacement was completely stopped. He has since been off steroids for 2 years, without any further recurrences of hypercalcemia, or Addisonian crisis since his initial presentation.

## 3 Discussion

Hypercalcemia, although reported to occur in adrenal insufficiency, is generally uncommon with only 5.5% of patients with primary adrenal insufficiency reported to have hypercalcemia (3) and also seen in patients with secondary adrenal insufficiency. Hypercalcemia in adrenal insufficiency is usually mild to moderate, with the severe hypercalcemia in our patient highly unusual. To our knowledge, this is the first case of severe hypercalcemia occurring due to adrenal insufficiency precipitated by fluconazole use.

### 3.1 Mechanism of hypercalcemia in adrenal insufficiency

Various mechanisms on how adrenal insufficiency can lead to hypercalcemia have been postulated, including decreased renal calcium excretion, increased bone resorption and less likely, increased gut calcium absorption. In adrenal insufficiency, there is volume depletion and reduced cardiac contractility, resulting in decreased glomerular filtration rate and reduction in the amount of calcium filtered. Coupled with increased calcium and sodium reabsorption at the proximal tubules, this results in decrease in calcium clearance (4, 5). Patients with primary AI are at greater risk due to concomitant mineralocorticoid deficiency and consequent volume depletion.

Hypercalcemia from increased bone resorption in adrenal insufficiency appears to be mediated by thyroid hormone. In adrenal insufficiency, loss of inhibition of pituitary TSH secretion by glucocorticoid may increase bone resorption and mobilize calcium from bone (6). Thyroid hormone replacement may precipitate hypercalcemic crisis in untreated Addison’s disease, possibly through increase cortisol clearance and increase bone resorption (7). In contrast, coexistence of secondary hypothyroidism in hypopituitarism appears to be protective against development of hypercalcemia (8). Our patient was euthyroid, which could have facilitated the development of hypercalcemia. Physiological amount of glucocorticoid may also be necessary for the acquisition and preservation of the differentiated state of osteoblasts (9).

Glucocorticoid inhibits 1α-hydroxylation of vitamin D; hence, there is a consideration whether the lack of cortisol promotes this process and results in increased gut calcium absorption. However, 1,25-hydroxyvitamin D levels have been found to be low in previous reports (5, 10). Calcium-free diet in animal studies and changes in dietary calcium intake did not alter the incidence or degree of hypercalcemia (5, 7), making this mechanism less likely.

### 3.2 Management of severe hypercalcemia in adrenal insufficiency

Severe hypercalcemia can lead to profound hypovolemia *via* gastrointestinal loss, hypercalcemia-induced diuresis, and nephrogenic diabetes insipidus. This is aggravated by the underlying adrenal insufficiency and profound shock, leading to multiorgan failure. Prompt treatment to lower calcium level, glucocorticoid replacement, and close monitoring for complications are required. Hydration with IV fluids corrects volume depletion and increases glomerular filtration rate and calciuresis when sodium is reabsorbed in exchange of calcium at the proximal tubules (11, 12). Forced calciuresis with loop diuretics is not routinely recommended as it can worsen volume depletion and other electrolytes abnormalities (11). Whereas aggressive volume repletion may improve hypercalcemia, calcium level only normalizes with adequate glucocorticoid replacement therapy, as evident in our patient and previous reports (5, 6, 13).

Drugs that inhibit osteoclastic-mediated bone resorption such as calcitonin, IV bisphosphonates (6, 14, 15), and denosumab can be used to temporarily lower calcium level while adequate glucocorticoid replacement takes effect. Calcitonin has the additional effect of increasing renal calcium excretion, and its rapid onset of action can be beneficial in severe hypercalcemia, but its use is limited due to tachyphylaxis and modest calcium-lowering effect. IV bisphosphonates are more potent in calcium-lowering but are relatively contraindicated in renal impairment due to risk of glomerular sclerosis and acute tubular necrosis (16). Denosumab, which is not renally cleared, can be considered in patients with renal impairment or bisphosphonate-resistant hypercalcemia (17). However, we did not find any reports on denosumab use in hypercalcemia secondary to adrenal insufficiency thus far. Hemodialysis is the most effective and rapid means of improving severe hypercalcemia. It can reduce calcium level by 0.75 to 1.25 mmol/L within hours and should be considered in patients with advanced oliguric renal failure or refractory hypercalcemia (11). It effectively lowered our patient’s calcium level but rebounded as glucocorticoid was withdrawn. Our case demonstrated the importance of treating the underlying adrenal insufficiency for resolution of hypercalcemia.

### 3.3 Fluconazole and adrenal insufficiency

Chronic exogenous steroid use is the commonest cause of adrenal suppression. Even if initial history was unyielding, surreptitious use of steroid in various preparation, including traditional medication, must be sought after. Our patient did not exhibit Cushingoid features. His HPA axis had likely been suppressed for a prolonged period, and the recent use of fluconazole precipitated the adrenal crisis.

Fluconazole is a triazole antifungal with greater affinity for fungal cytochrome P450 compared to mammalian cytochrome P450, making it less toxic and favorable compared to ketoconazole in treatment of fungal infection (18). Ketoconazole is an imidazole antifungal that inhibits side-chain cleavage, 17,20-lyase, and 11β-hydroxylase enzymes in adrenal steroidogenesis pathway and is also used in the treatment of Cushing’s syndrome (19). However, the role of fluconazole in affecting steroidogenesis is less clear.

Fluconazole was found to inhibit 11β-hydroxylase and 17-hydroxylase activity in pharmacological doses *in vitro*. Its inhibitory effect on cortisol production was dose-dependent and less potent compared to ketoconazole (20). Reduction in cortisol level has been observed with low doses of fluconazole of 200 mg daily (21, 22). In a study examining the use of fluconazole of 400 mg daily in critically ill patients, basal ACTH, basal cortisol, and ACTH-stimulated cortisol levels were not significantly altered (23). Similarly, fluconazole prophylaxis in critically ill surgical patients did not lower the median plasma cortisol level or result in adrenal dysfunction (24). However, several case reports have described acute adrenal insufficiency in patients receiving fluconazole, in both critically ill and stable patients, which was reversible after cessation of anti-fungal (**Table 1**). Some of these patients had other precipitating factors for adrenal insufficiency such as previous use of dexamethasone (21, 25), megestrol (25), or combination of ritonavir and fluticasone (26).

**Table 1:** Case reports on adrenal insufficiency with triazole antifungals use and the associated calcium level.

	Age/gender	Antifungal implicated	Evaluation for adrenal insufficiency (AI)	Type of AI	Calcium level	Other relevant history/precipitants	Outcome
**Albert et al., 2001** (25)	66, female	Fluconazole of 200–400 mg daily	SST before fluconazole: * B. cortisol of 516 nmol/L * P. cortisol of 773 nmol/L SST after 9 days on fluconazole: * B. cortisol of 408 nmol/L * P. cortisol of 464 nmol/L No ACTH values	Unknown	Not reported	Admitted for septic shock secondary to staphylococcus aureus bacteremia and left knee septic arthritis	Normalization of peak SST response 4 days after stopping Fluconazole: * B. cortisol of 499 nmol/L * P. cortisol of 568 nmol/L * ACTH of 60 ng/L
**Shibata et al., 2001** (21)	63, male	Fluconazole of 200 mg daily (days 1–8 of admission) From day 16, Fluconazole restarted at 400 mg daily	SST after 8 days on fluconazole (200 mg daily): * B. cortisol of 276 nmol/L * P. cortisol of 662 nmol/L Patient rechallenged with fluconazole (400 mg daily). SST after 5 days of restarting: * B. cortisol of 166 nmol/L * P. cortisol of 414 nmol/L No ACTH values	Unknown	Not reported	Multiple myeloma Received VAD regimen (vincristine, doxorubicin, and dexamethasone) Underwent peripheral stem cell harvesting after receiving high dose cyclophosphamide	Continued on fluconazole prophylaxis with concurrent corticosteroid supplementation
**Krishnan et al.,** **2006** (18)	38, male	Fluconazole of 400 mg daily	SST before fluconazole: * P. cortisol of 663 nmol/L SST after 2 days on fluconazole: * B. cortisol of 45 nmol/L * P. cortisol of 375 nmol/L No ACTH values	Unknown	Not reported	Hypercapnic respiratory failure secondary to pneumonia	Normalization of peak SST response 10 days after stopping fluconazole: * B. cortisol of 38 nmol/L * P. cortisol of 662 nmol/L
**St Clair et al.,** **2012** (26)	52, male	Fluconazole of 400 mg daily Inhaled fluticasone was stopped concurrently	SST less than 1 week after starting fluconazole and stopping inhaled fluticasone: * B. cortisol of 47 nmol/L * P. cortisol of 353 nmol/L * ACTH of <5 ng/L	SAI (possibly potentiation of fluticasone effect due to ritonavir), aggravated by PAI induced by fluconazole	Not reported	On ritonavir, atazanavir and efavirenz for HIV infection On inhaled fluticasone propionate (250 mg twice daily) for chronic bronchitis	No follow up of the HPA axis reported
**Freyer et al., 2022** (27)	70, female	Fluconazole of 400 mg daily	SST after 67 days on fluconazole * B. cortisol of 116 nmol/L * P. cortisol of 353 nmol/L * ACTH of 96.7 ng/L	PAI	Not reported	B-cell acute lymphoblastic leukemia, underwent allogenic hematopoietic cell transplantation	Fluconazole was initially stopped and switched to caspofungin. Fluconazole was later restarted with concurrent hydrocortisone supplementation. SST 31 days after fluconazole was restarted: * B. cortisol of 268 nmol/L * P. cortisol of 339 nmol/L * ACTH of 123.5 ng/L
**Miller et al.,** **2017** (28)	63, male	Posaconazole of 300 mg daily	SST after 2 months on posaconazole: * B. cortisol of 52 nmol/L * P. cortisol of 113 nmol/L * ACTH of 154.6 ng/L	PAI	2.13 mmol/L	Chronic myelomonocytic leukemia Had intermittent steroid injections into his spine. Last steroid injection 3 months before presentation	Normalization of peak SST response 1 year after stopping posaconazole: * B. cortisol of 138 nmol/L * P. cortisol of 510 nmol/L * ACTH of 34.1 ng/L
**Araque et al., 2020** (29)	65, male	Posaconazole of 500 mg daily	Blood tests done on dexamethasone of 3 mg daily and indeterminate duration of posaconazole: * B. cortisol of 11 nmol/L * ACTH of 3.4 ng/L * Sodium of 130 mmol/L * Potassium of 5.1mmol/L * Renin 16.7 of ng/ml/h * Aldosterone of 1.6 ng/dl	PAI induced by posaconazole, on background of SAI from dexamethasone	Not reported	Hemophagocytic lymphohistiocytosis on dexamethasone	One month after stopping posaconazole, B. cortisol, and ACTH showed recovering HPA axis * B. cortisol of 359 nmol/L * ACTH of 53.9 ng/L
**Villar-Prados et al., 2021** (30)	56, male	Posaconazole of 300 mg daily Patient was subsequently switched to fluconazole of 800 mg daily	SST after 5 months on Posaconazole: * B. cortisol of 160 nmol/L * P. cortisol of 198 nmol/L * ACTH of 168 ng/L	PAI	Not reported	Chronic disseminated coccidioidomycosis	SST after 7 months on Fluconazole showed persistent hypocortisolism: * B. cortisol of 5.5 nmol/L * P. cortisol of 22 nmol/L * ACTH of 34 ng/L * Renin of 2.6 ng/ml/h * Aldosterone of <4.0 ng/dl
**Nalla et al.,** **2017** (31)	72, female	Itraconazole of 200 mg daily	Blood tests done after 2 years of itraconazole: * Random cortisol of 4 nmol/L * ACTH of <10 ng/L * MRI pituitary normal	SAI (possibly potentiation of fluticasone effect due to itraconazole)	Not reported	Allergic bronchopulmonary aspergillosis On Seretide evohaler (fluticasone of 500 µg and salmeterol of 50 μg) daily for asthma	No follow up of the HPA axis reported

PAI, primary adrenal insufficiency; SAI, secondary adrenal insufficiency; HPA, hypothalamic-pituitary-adrenal axis; ACTH, adrenocorticotropic hormone; B. cortisol, basal (non-stimulated) cortisol; P. cortisol, peak cortisol response after 250 mcg of Synacthen (cosyntropin) stimulation test; SST, 250 µg of Synacthen (Cosyntropin) stimulation test.

In most of these case reports, adrenal insufficiency occurred from 24 h to 68 days after initiation of the drug (25, 32). Most cases of adrenal suppression were reversible within 5 to 11 days after cessation of fluconazole (18, 21, 25). Unlike the previous cases, our patient remained hypocortisolic for 8 years after stopping fluconazole, which was likely due to prolonged suppression of the HPA axis from exogenous steroid use. HPA axis recovery is known to vary widely due to interindividual susceptibility, dose, and duration of steroid exposure, with recovery in weeks to years, or may not occur at all in some patients (33). Concurrent use of fluconazole during dexamethasone therapy was associated with prolonged HPA recovery (34). This is due to the inhibition of CYP3A4 by fluconazole, which results in decreased metabolism of corticosteroid and increased corticosteroid exposure to the HPA axis (35). Itraconazole is an even more potent inhibitor of CYP3A4, and secondary adrenal insufficiency was reported to occur in a patient on inhaled fluticasone and itraconazole (31). Another triazole antifungal, posaconazole, has also been reported to lead to adrenal insufficiency in two case reports (28, 30).

There have been case reports of hypercalcemia occurring with azole antifungal use, although cortisol levels were not measured, and it is unclear whether hypocortisolism may have contributed to hypercalcemia (36, 37). The authors postulated that hypercalcemia may have occurred due to inhibition of CYP enzymes responsible for the metabolism of vitamin A or *via* PTH-mediated hypercalcemia. Interestingly, fluconazole has also been reported to be used to treat hypercalcemia by inhibiting 1α-hydroxylase. This was used in patients with mutations of CYP24A1 and SLC34A3 gene, leading to persistently raised 1,25-dihydroxyvitamin D, hypercalcemia, hypercalciuria, and nephrocalcinosis/nephrolithiasis (38, 39).

### 3.4 Conclusion

Adrenal insufficiency is a rare but important cause of severe hypercalcemia. Both adrenal insufficiency and hypercalcemia have similar non-specific symptoms, including anorexia, vomiting, postural giddiness, confusion, and hypotension. Failure to assess cortisol status or serum calcium levels may lead to a delay in diagnosis with fatal consequences. In patients with hypercalcemia of unknown etiology, adrenal insufficiency should be considered. The most common cause of adrenal insufficiency remains exogenous steroid use, and a careful review of drug history is warranted. Finally, although not as potent as ketoconazole, physicians should be mindful of the risk of hypocortisolism when initiating patients on triazole antifungals, which may precipitate an Addisonian crisis.

## Author contributions

TP and KC were involved in conception of the manuscript. KC wrote the first draft, and JY and TP wrote additional sections of the manuscript. ET and TP were involved in the clinical care of the patient. All authors contributed to the article and approved the submitted version.
